# Exogenous Metabolic Modulators Improve Response to Carboplatin in Triple-Negative Breast Cancer

**DOI:** 10.3390/cells13100806

**Published:** 2024-05-09

**Authors:** Alyssa N. Ho, Violet A. Kiesel, Claire E. Gates, Bennett H. Brosnan, Scott P. Connelly, Elaine M. Glenny, Alyssa J. Cozzo, Stephen D. Hursting, Michael Francis Coleman

**Affiliations:** 1 Department of Nutrition, University of North Carolina at Chapel Hill, Chapel Hill, NC 27599, USA; 2 Department of Pharmacology, University of North Carolina at Chapel Hill, Chapel Hill, NC 27599, USA; 3 Nutrition Research Institute, University of North Carolina at Chapel Hill, Kannapolis, NC 28081, USA; 4 Lineberger Comprehensive Cancer Center, University of North Carolina at Chapel Hill, Chapel Hill, NC 27599, USA

**Keywords:** triple-negative breast cancer, chemotherapy, combination therapy, metabolism, insulin receptor/insulin-like growth factor 1 receptor, lysosomes, autophagy

## Abstract

Triple-negative breast cancer (TNBC) lacks targeted therapies, leaving cytotoxic chemotherapy as the current standard treatment. However, chemotherapy resistance remains a major clinical challenge. Increased insulin-like growth factor 1 signaling can potently blunt chemotherapy response, and lysosomal processes including the nutrient scavenging pathway autophagy can enable cancer cells to evade chemotherapy-mediated cell death. Thus, we tested whether inhibition of insulin receptor/insulin-like growth factor 1 receptor with the drug BMS-754807 and/or lysosomal disruption with hydroxychloroquine (HCQ) could sensitize TNBC cells to the chemotherapy drug carboplatin. Using in vitro studies in multiple TNBC cell lines, in concert with in vivo studies employing a murine syngeneic orthotopic transplant model of TNBC, we show that BMS-754807 and HCQ each sensitized TNBC cells and tumors to carboplatin and reveal that exogenous metabolic modulators may work synergistically with carboplatin as indicated by Bliss analysis. Additionally, we demonstrate the lack of overt in vivo toxicity with our combination regimens and, therefore, propose that metabolic targeting of TNBC may be a safe and effective strategy to increase sensitivity to chemotherapy. Thus, we conclude that the use of exogenous metabolic modulators, such as BMS-754807 or HCQ, in combination with chemotherapy warrants additional study as a strategy to improve therapeutic responses in women with TNBC.

## 1. Introduction

Breast cancer (BC) was the second leading cause of cancer death among women in the United States in 2023 [1]. Approximately 30% of women diagnosed with early-stage BC will develop metastases, and their five-year survival rate will drop from 85–99% to 25%, with a median overall survival of approximately 24 months [2]. Triple-negative breast cancer (TNBC) accounts for 10–20% of invasive BC cases and is defined by the absence of estrogen receptor (ER), progesterone receptor (PR), and human epidermal growth factor receptor 2 (HER2) [3]. TNBC is associated with a higher risk of recurrence and metastasis compared with other BC subtypes [3,4].

As TNBC lacks ER, PR, and HER2, few targeted therapies are available to patients. For recurrent or metastatic TNBC, the preferred first-line treatment is the programmed cell death protein 1 inhibitor, pembrolizumab, plus chemotherapy, including carboplatin (CP), paclitaxel, or gemcitabine [5]. However, for the treatment of early-stage TNBC, pembrolizumab is still under investigation, and neoadjuvant poly-chemotherapy remains the standard of care [6]. Platinum-based alkylating chemotherapy agents (e.g., CP and cisplatin) induce double-stranded breaks (DSBs) in DNA in both normal and cancer cells [3,7]. In normal cells, DSBs activate the DNA damage response to repair the damaged DNA and maintain genomic stability [8,9,10]. In contrast, cancer cells have a dysregulated DNA damage response; thus, CP-induced DSBs inhibit DNA replication and transcription, ultimately resulting in cancer cell apoptosis and tumor shrinkage [8,9,10]. Sensitizing agents that increase the efficacy of CP have the potential to reduce both chemotherapy resistance and the cumulative CP dose.

In the so-called TNBC “paradox”, patients with TNBC, relative to other BC subtypes, are typically more responsive to preoperative chemotherapy yet have a higher rate of overall relapse [11]. Several mechanisms mediate resistance of cancer cells to DNA-damaging agents, including enhanced drug efflux via ATP-binding cassette transporters, drug inactivation via molecular or metabolic alterations, reduced drug absorption, enhanced DNA repair, and lysosome-mediated mechanisms including autophagy [9,12,13,14,15]. Autophagy is a highly conserved process in which cellular contents, including damaged organelles, are degraded to provide metabolic substrates to cells, thus promoting cell survival by eliminating cellular signals that would ordinarily induce apoptosis [16,17,18,19]. To combat chemotherapy resistance, CP is often combined with additional chemotherapy drugs, an approach that can be more effective than single-agent treatments [5,20]. Despite these combinatorial approaches, patients with TNBC residual disease following neoadjuvant systemic therapy still face poor prognosis, accentuating the need for novel strategies to minimize chemotherapy resistance and inhibit TNBC progression.

Metabolic dysregulation is a hallmark of cancer that represents a promising target for combinatorial treatment of TNBC [21,22]. Signaling through insulin receptor (IR) and insulin-like growth factor 1 receptor (IGF-1R) activates the phosphatidylinositol 3-kinase/RAC-alpha serine/threonine-protein kinase/mammalian target of rapamycin (PI3K/AKT/mTOR) pathway. Activation of this pathway plays a critical role in regulating cell metabolism and promoting cell proliferation and survival, which are processes involved in chemotherapy resistance [22,23]. Indeed, IGF-1R signaling through the PI3K/AKT/mTOR cascade promotes chemotherapeutic resistance in ovarian, breast, prostate, and bladder cancers [24]. To date, when used alone, inhibitors targeting IR/IGF-1R have shown limited anticancer efficacy in clinical trials [25,26]. For example, in a phase II trial in women with hormone receptor-positive BC, therapy with the IGF-1R antibody cixutumumab failed to enable progression-free survival >4.4 months, which was the primary objective based upon the historical standard obtained with fulvestrant; further investigation was not pursued [25]. In another phase II trial, progression-free survival in patients with non-small cell lung cancer was lower when treated with the IGF-1R signaling inhibitor AXL1717 than docetaxel [26]. Though single-agent use of IGF-1R inhibitors has demonstrated little benefit, combining these inhibitors with chemotherapies may be an effective strategy for increasing therapeutic response.

Collectively, both lysosomal disruption and IR/IGF-1R inhibition may improve response to chemotherapy. Furthermore, autophagy is negatively regulated by mTOR activation [27], suggesting that inhibiting IR/IGF-1R may upregulate autophagy by limiting PI3K/AKT/mTOR activation to drive autophagic flux. We investigated whether disrupting lysosomal dynamics, alone or in combination with an IR/IGF-1R inhibitor, could sensitize cancer cells to chemotherapy. Specifically, we interrogated combination regimens with the irreversible platinum-based chemotherapy drug CP, the reversible dual IR/IGF-1R inhibitor BMS-754807, and an inhibitor of lysosomal function (hydroxychloroquine, HCQ) in both in vitro and in vivo models of TNBC. We hypothesized that exogeneous metabolic modulators such as BMS-754807 or HCQ would sensitize TNBC cells and tumors to CP.

## 2. Materials and Methods

### 2.1. Chemicals and Reagents

BMS-754807 was purchased from MedChemExpress (Monmouth Junction, NJ, USA), CP from Accord (Durham, NC, USA), and recombinant human IGF-1 from PeproTech (Cranbury, NJ, USA). Protease inhibitor cocktail, sodium pyrophosphate, and 3-(4,5-dimethylthiazol-2-yl)-2,5-diphenyltetrazolium bromide (MTT) reagent were purchased from ThermoFisher (Waltham, MA, USA). Hydroxychloroquine sulfate was purchased from Tokyo Chemical Industry (Portland, MA, USA). Sodium orthovanadate, β-glycerophosphate, isopropyl β-D-1-thiogalactopyranoside (IPTG), rapamycin, bafilomycin A1, and hygromycin were purchased from Sigma Aldrich (St. Louis, MO, USA). PMD2.G and psPAX2 packaging plasmids were purchased from Addgene (Watertown, MA, USA). ATG5 targeting (MSH091034-LVRU6H) and scramble control (CSHCTR001-LVRU6H) shRNA vectors were purchased from Vectorbuilder (Chicago, IL, USA).

### 2.2. Cell Culture

E0771, MDA-MB-231, and MDA-MB-468 cells were purchased from American Type Culture Collection (Manassas, VA, USA). metM-Wnt^lung^ cells were derived from serial transplantation of nonmetastatic M-Wnt murine mammary cancer cells through five generations of severe-combined immunodeficient mice and display increased metastatic potential to the lung when orthotopically injected into C57BL/6 mice [28]. B6C3TAg 1.02, 2.03, and 2.51 TNBC cell lines were derived from 10 generations of C3TAg transgenic FVB mice backcrossed with C57BL/6 mice whose spontaneous mammary tumors were then dissociated and subcloned [29]. Murine E0771 and metM-Wnt^lung^ mammary cancer cells and human MDA-MB-231 and MDA-MB-468 breast cancer cells were cultured in Dulbecco’s Modified Eagle Medium (DMEM, Gibco, Waltham, MA, USA) containing 4.5 g/L glucose. Murine B6C3TAg 1.02, 2.03, and 2.51 mammary cancer cells were cultured in RPMI 1640 (Gibco, Waltham, MA, USA). For experiments, cells were seeded in 1 g/L glucose DMEM to investigate therapeutic response under conditions consistent with homeostatic glucose levels in a normoglycemic individual. All media were supplemented with 10% fetal bovine serum (Avantor, Radnor Township, PA, USA) and 2 mM L-glutamine (Gibco, Waltham, MA, USA). Cells were maintained at 37 °C in a humidified 5% CO_2_ chamber.

### 2.3. Generation of Inducible shATG5 Cell Lines

HEK293T cells were transfected with PMD2.G and psPAX2 packaging plasmids and IPTG-inducible shRNA expression constructs (ATG5-targeted (MSH091034-LVRU6H) or non-targeted scramble control (CSHCTR001-LVRU6H)) to generate lentiviral particles. Lentiviral particles were then used to transduce B6C3TAg 1.02 and 2.51 TNBC cell lines. Following selection for hygromycin resistance for 7 d, shRNA expression was induced by treating cells with 1 mM IPTG for 48 h.

### 2.4. Western Immunoblotting

To analyze protein from cultured cells, cells were seeded in 1 g/L glucose DMEM and incubated overnight. For serum-containing conditions, cells were seeded overnight in low-glucose medium and treated with vehicle (DMSO, 2.5 µM Sigma Aldrich, St. Louis, MO, USA) or BMS-754807 (2.5 µM) for 4 h. For serum-free conditions, cells were seeded overnight in serum-containing 1 g/L glucose medium and then treated for 3 h in serum-free medium followed by 1 h of treatment with vehicle (DMSO, 2.5 µM), IGF-1 (10 ng/mL), BMS-754807 (2.5 µM), or a combination of BMS-754807 + IGF-1. Protein was isolated using radioimmunoprecipitation buffer supplemented with protease inhibitor cocktail (1%) and phosphatase inhibitors (sodium orthovanadate, sodium pyrophosphate, and β-glycerophosphate, 1% each). Cell debris was pelleted by centrifugation at 24,000× *g* for 15 min at 4 °C, and supernatants were retained. Bradford assay (BioRad, Hercules, CA, USA) was performed to determine protein concentration. Equal amounts of protein were separated via SDS-PAGE and transferred to nitrocellulose membrane (Bio-Rad). Membranes were blocked with 5% bovine serum albumin in tris-buffered saline with 0.1% tween before incubating overnight at 4 °C with primary antibody. Membranes were then incubated with secondary IRDye 680 RD goat anti-mouse (LI-COR #926-68070, 1:10,000) or IRDye 800CW goat anti-rabbit (LI-COR #926-32211, 1:10,000) antibody. Antibody binding was detected with the Odyssey Imaging System (LI-COR, Lincoln, NE, USA). Images were analyzed via near-infrared fluorescence using Image Studio Lite software (version 5.2.5, LI-COR Biosciences, Lincoln, NE, USA). The following primary antibodies were used: p-AKT S473 (Cell Signaling Technology (CST, Danvers, MA, USA) #4060S, 1:1000), AKT (CST #9272S, 1:1000), p-S6 Ser 235/236 (CST #2211S, 1:1000), S6 (CST #2317, 1:1000), ATG5 (CST #2630S, 1:1000), LC3 (CST #2775S, 1:1000), α-tubulin (Santa Cruz Biotechnology (Dallas, TX, USA, #sc-398103, 1:5000), and β-actin (Santa Cruz Biotechnology #sc-47778, 1:5000). All experiments were performed with *n* ≥ 3.

### 2.5. Autophagy Measurement

To assay autophagy induction, cells were seeded overnight in low-glucose media, treated with BMS-754807 (2.5 µM) or rapamycin (1 μM) for 4 h, then treated with or without bafilomycin A1 (200 nM) for 4 h. Accumulation of LC3-II was determined by Western blot and expressed as relative to control. 

### 2.6. Viability Assays 

Cells were seeded in 96-well plates in 1 g/L glucose DMEM overnight. In single-agent experiments (BMS-754807 only), cells were treated with BMS-754807 (0–10 μM) for 24 h. In two-drug combination experiments, medium containing BMS-754807 (0–10 μM) or HCQ (0–40 μM) was added to cells for 24 h. Cells were then treated with fresh medium containing the same drug (either BMS-754807, 0–10 μM; or HCQ, 0–40 μM), with either CP (0–400 μg/mL) or IPTG (to induce shRNA expression) for 48 h. The highest concentration of solvent achieved was 0.1% DMSO. In three-drug combination experiments, medium containing BMS-754807 (0–1.25 μM) was added to cells for 24 h. Cells were then treated with fresh medium containing BMS-754807 (0–1.25 μM), CP (0–200 μg/mL), and HCQ (0–30 μM) for 48 h. After treatment in each experiment type, medium was replaced with 0.5 mg/mL MTT in serum-free medium. Crystals were dissolved in 100% DMSO and quantified by absorbance measurements at 570 and 690 nm (Cytation 3 Cell Imaging Reader, Biotek, Winooski, VT, USA). CP concentrations were selected to be similar to maximum (C_max_) concentrations in human plasma after administration of 150–450 mg/m^2^ CP (~12.5–50 μg/mL) [30].

### 2.7. Animal Study Design

Female C57BL/6NCrl mice (*n* = 120) were purchased from Charles River (Wilmington, MA, USA) and allowed to acclimate to a low-fat control diet (10 kcal% from fat, Research Diets D12450J) ad libitum. At 36 weeks of age, mice were orthotopically injected into the fourth mammary fat pad with 50,000 metM-Wnt^lung^ mammary cancer cells in 50 μL sterile phosphate-buffered saline. Tumors grew until reaching a mean size of 100 mm^3^, at which time mice were block randomized to receive either vehicle, BMS-754807 (6.25 mg/kg), HCQ (60 mg/kg), CP (50 mg/kg), or combinations of two or three drugs. Doses used for in vivo analysis were based on the previous literature [31,32,33,34,35,36]. BMS-754807 and/or HCQ were delivered by daily intraperitoneal injection in a vehicle of 30% PEG300, 5% DMSO, 5% Tween80, and 60% water. CP dissolved in water was delivered by once-weekly intraperitoneal injection. After 23 d of drug treatment (1–2 d prior to euthanasia), blood glucose was assessed via tail nick and portable glucometer (Bayer, Pittsburgh, PA, USA). Blood glucose assessment was performed 2 h and 6 h after drug treatment. Tumor sizes were monitored three times a week until average tumor size in the largest treatment group reached a volume of 1250 mm^3^, at which time all mice were euthanized, and tumors were excised and weighed.

## 3. Results

### 3.1. BMS-754807 Is Cytotoxic in TNBC Cells

**Figure 1:**
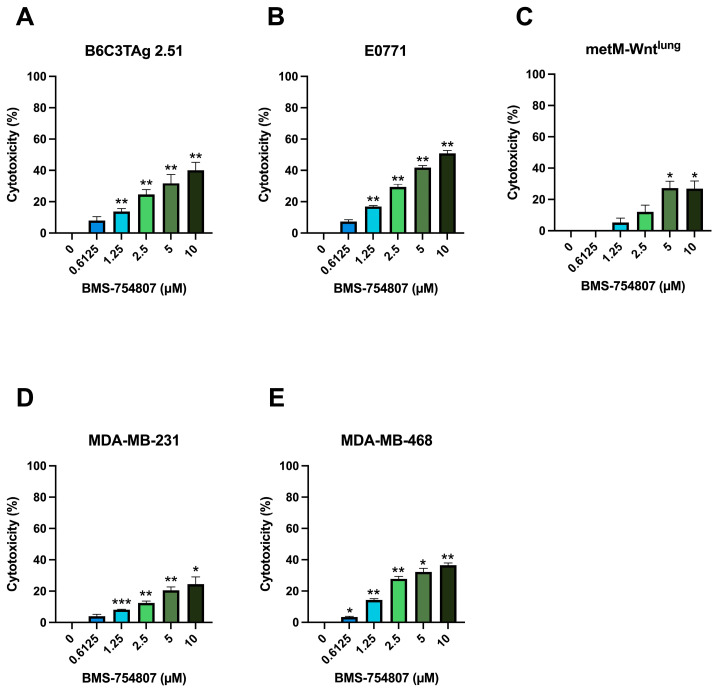
Effect of BMS-754807 treatment on cytotoxicity in TNBC cells. Cytotoxicity following 24 h treatment with BMS-754807 was assessed by MTT assay in (**A**) B6C3TAg 2.51; (**B**) E0771; (**C**) metM-Wnt^lung^; (**D**) MDA-MB-231; and (**E**) MDA-MB-468 TNBC cell lines. Data presented as mean ± SEM for *n* = 3 (E0771, MDA-MB-468), *n* = 4 (MDA-MB-231), *n* = 5 (metM-Wnt^lung^), and *n* = 6 (B6C3TAg 2.51) experiments. Asterisks denote significance: * *p* < 0.05, ** *p* < 0.01, *** *p* < 0.001 versus vehicle by one-way ANOVA followed by Dunnett’s post hoc test.

We first interrogated whether murine and human TNBC cells were sensitive to inhibition of IR/IGF-1R signaling by treating with BMS-754807 (0–10 μM) for 24 h before assessing cellular viability by MTT assay. In all five cell lines tested, a dose-dependent increase in cytotoxicity was observed in response to BMS-754807, and the highest dose of BMS-754807 significantly induced 27–51% cytotoxicity compared with vehicle treatment (Figure 1A–E).

**Figure 2:**
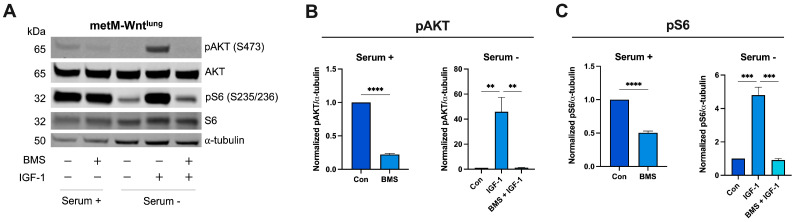
Effect of BMS-754807 on protein expression downstream of insulin receptor (IR)/insulin-like growth factor 1 receptor (IGF-1R). Representative Western blots from (**A**) metM-Wnt^lung^ cells; quantification of (**B**) pAKT, relative to AKT and alpha-tubulin; and (**C**) pS6, relative to S6 and alpha-tubulin. Data are presented as mean ± SEM from *n* = 3 experimental replicates. Con group represents cells treated with vehicle (DMSO, 2.5 µM). Differences between two groups were analyzed using unpaired *t*-test; differences across groups were analyzed using one-way ANOVA, followed by Tukey’s post hoc test. Asterisks denote significance: ** *p* < 0.01, *** *p* < 0.001, **** *p* < 0.0001. Serum + indicates 1 g/L glucose DMEM + 10% FBS. Serum—indicates 1 g/L glucose DMEM without FBS.

We next confirmed that treatment with BMS-754807 effectively inhibited its anticipated targets by examining protein phosphorylation downstream of IR/IGF-1R activation (Figure 2A). In serum-containing medium, BMS-754807 treatment (2.5 μM) significantly reduced levels of pAKT by 78% and pS6 by 50%, compared with vehicle, in metM-Wnt^lung^ cells (Figure 2B,C). In serum-free conditions, stimulation with 10 ng/mL IGF-1 increased levels of pAKT and pS6, which were reduced to baseline levels by co-treatment with IGF-1 and BMS-754807 (Figure 2B,C). Similar results were found in B6C3TAg 2.51 and E0771 cells (Figure S1).

### 3.2. Pretreatment with BMS-754807 Sensitizes TNBC Cells to CP

**Figure 3:**
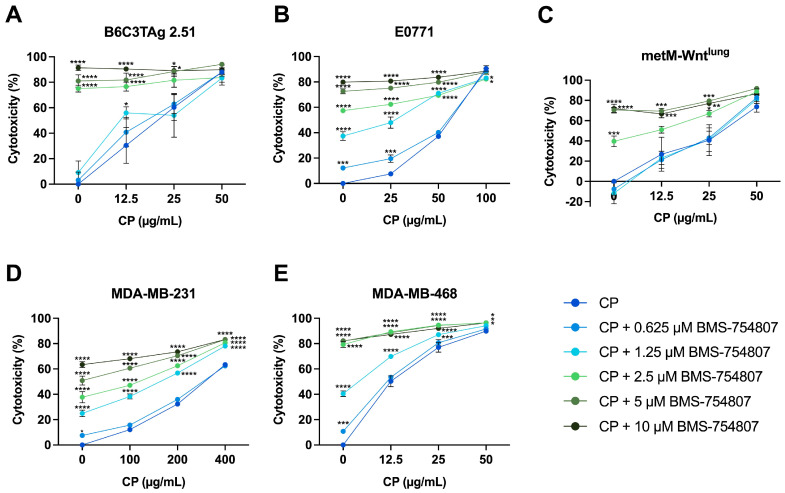
Effect of BMS-754807 and carboplatin (CP) combination treatment on cytotoxicity in TNBC cells. Cytotoxicity of BMS-754807 and CP combination treatment in (**A**) B6C3TAg 2.51, (**B**) E0771, (**C**) metM-Wnt^lung^, (**D**) MDA-MB-231, and (**E**) MDA-MB-468 TNBC cell lines was determined by MTT assay. Sensitivity to BMS-754807 alone is denoted by 0 μg/mL CP on each graph. Data presented as mean ± SEM for *n* = 3 (E0771, MDA-MB-231, MDA-MB-468) and *n* = 4 (B6C3TAg 2.51, metM-Wnt^lung^) experiments. Differences across groups were analyzed using two-way ANOVA, followed by Dunnett’s post hoc test. Asterisks denote significance: * *p* < 0.05, ** *p* < 0.01, *** *p* < 0.001, **** *p* < 0.0001 versus CP only.

TNBC cells were treated with BMS-754807 (0–10 μM) for 24 h, followed by combination treatment with CP (0–400 μg/mL) for 48 h to investigate the anticancer benefit of IR/IGF-1R inhibition prior to chemotherapy treatment. Combination treatment with BMS-754807 (5 μM) and CP (25 μg/mL) increased cytotoxicity by 28–68% compared with treatment with CP (25 μg/mL) alone in B6C3TAg 2.51 (*p* < 0.05), E0771 (*p* < 0.0001), metM-Wnt^lung^ (*p* < 0.001), and MDA-MB-468 (*p* < 0.0001) cells (Figure 3A–C,E). In MDA-MB-231 cells, combination treatment with BMS-754807 (1.25 μM) and CP (200 μg/mL) increased cytotoxicity compared with treatment with CP alone (200 μg/mL) (*p* < 0.0001) (Figure 3D).

Since these results indicated that BMS-754807 enhanced the response to CP, we next evaluated the potential for synergy between BMS-754807 and CP using the Bliss independence model and calculated synergy scores for all combinations of BMS-754807 and CP. Though there is no universal threshold to define synergy, we and others define a score >10 as indicating a likely synergistic interaction between two drugs [37]. The combination of BMS-754807 and CP produced synergy scores >10 in all cell lines, and Table 1 displays both the highest synergy score and the synergy score that resulted in ~50% cytotoxicity per cell line. Of the cell lines tested, B6C3TAg 2.51 showed the highest synergy score (34.71) between BMS-754807 and CP (Table 1).

### 3.3. Pretreatment with HCQ Sensitizes TNBC Cells to CP

**Figure 4:**
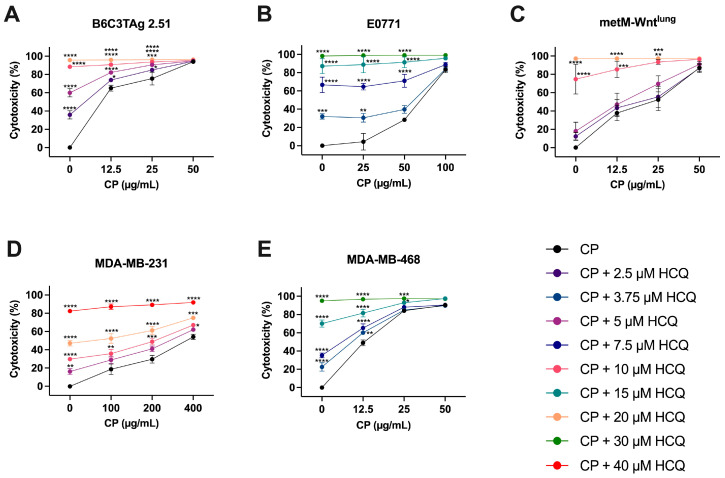
Effect of HCQ and CP combination treatment on cytotoxicity in TNBC cells. Cytotoxicity of HCQ and CP combination treatment was assessed in (**A**) B6C3TAg 2.51; (**B**) E0771; (**C**) metM-Wnt^lung^; (**D**) MDA-MB-231; and (**E**) MDA-MB-468 TNBC cell lines by MTT assay. Sensitivity to HCQ alone is denoted by 0 μg/mL CP on each graph. Data presented as mean ± SEM for *n* = 3 (B6C3TAg 2.51, E0771, MDA-MB-231, MDA-MB-468) and *n* = 4 (metM-Wnt^lung^) experiments. Differences across groups were analyzed using two-way ANOVA, followed by Dunnett’s post hoc test. Asterisks denote significance: * *p* < 0.05, ** *p* < 0.01, *** *p* < 0.001, **** *p* < 0.0001 versus CP only.

Upregulation of autophagy in cancer cells may mediate resistance to DNA-damaging chemotherapy [16,17,19]. We first investigated the effect of HCQ alone on TNBC cells and demonstrated HCQ-induced cytotoxicity in a panel of TNBC cell lines (Figure S2). We next evaluated the anticancer effect of HCQ in combination with CP. TNBC cells were first treated with HCQ (0–30 μM) for 24 h to disrupt lysosomal function before the addition of CP (0–400 μg/mL) for 48 h of combination treatment. Combination treatment with HCQ 10 μM) and CP (25 μg/mL) increased cytotoxicity by 18–41% compared with treatment with CP alone in B6C3TAg 2.51 (*p* < 0.0001) and metM-Wnt^lung^ (*p* < 0.01) cells (Figure 4A,B). Combination treatment with HCQ (15 μM) and CP (25 μg/mL) increased cytotoxicity by 9–84% compared with treatment with CP alone in E0771 (*p* < 0.0001) and MDA-MB-468 (*p* < 0.05) cells (Figure 4B,E). In MDA-MB-231 cells, combination treatment with HCQ (20 μM) and CP (100 μg/mL) increased cytotoxicity by 34% compared with treatment with CP (100 μg/mL) alone (*p* < 0.0001) (Figure 4D).

We again utilized Bliss independence to evaluate potential synergy between HCQ and CP treatment. In MDA-MB-231 and MDA-MB-468 cells, combination treatment resulted in synergy scores >10, suggesting that HCQ may synergize with CP in these human cell lines (Table 2). In the E0771, B6C3TAg 2.51, and metM-Wnt^lung^ murine cell lines, the synergy scores were between 0 and 10, suggesting at most an additive interaction between HCQ and CP (Table 2).

### 3.4. HCQ Treatment Sensitizes Viability of TNBC Cells to BMS-754807 and CP

BMS-754807 suppresses PI3K/AKT/mTOR downstream of IR/IGF-1R, thus sensitizing cancer cells to CP (Figure 2 and Figure 3) and potentially increasing autophagic flux through suppression of mTOR. To assay whether BMS-754807 induces autophagy, we performed LC3 immunoblotting. LC3 is lipidated as autophagy proceeds and is either degraded or recycled following cargo degradation. Thus, in the presence of an inhibitor of lysosomal acidification, LC3 accumulation can estimate induction of autophagic flux. BMS-754807 induced lipidation of LC3 as demonstrated by greater accumulation of LC3-II in BMS-754807-treated cells relative to control cells when lysosomal acidification was inhibited with V-ATPase inhibitor bafilomycin A1 (Figure S3).

**Figure 5:**
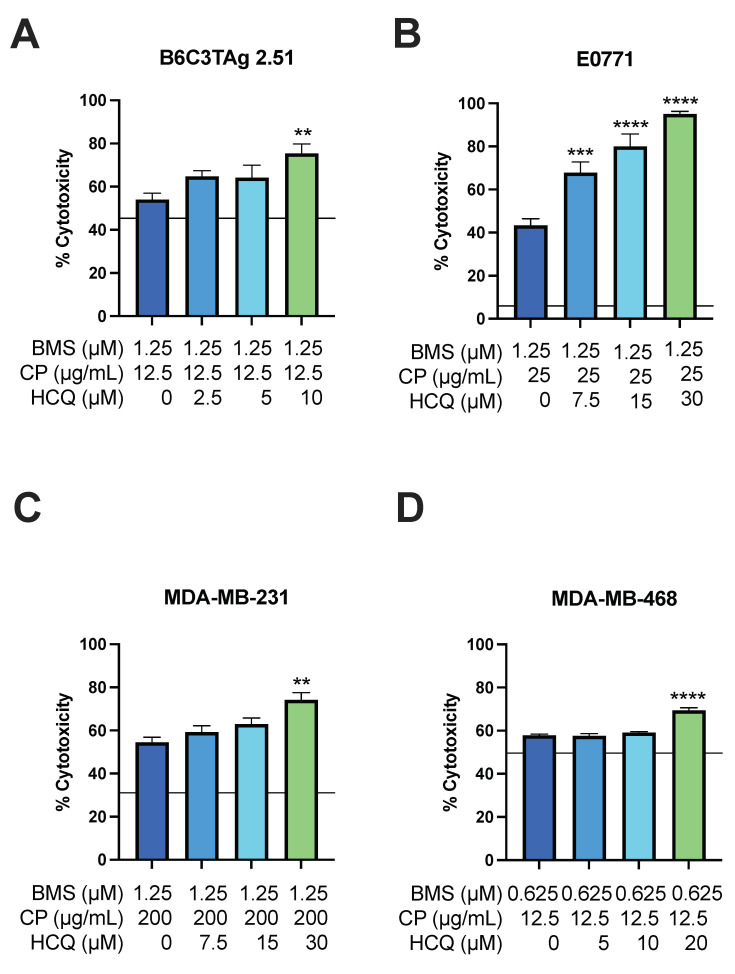
Effect of BMS-754807, CP, and HCQ triple treatment in TNBC cells. Cytotoxicity of BMS-754807, CP, and HCQ triple treatment was assessed in (**A**) B6C3TAg 2.51; (**B**) E0771; (**C**) MDA-MB-231; and (**D**) MDA-MB-468 TNBC cell lines by MTT assay. Data presented as mean ± SEM for *n* = 4 (MDA-MB-231, MDA-MB-468), *n* = 5 (B6C3TAg 2.51), and *n* = 9 (E0771) experiments. Horizontal line denotes average cytotoxicity elicited by indicated carboplatin concentration alone in Figure 3 and Figure 4. Differences between groups were analyzed using one-way ANOVA, followed by Dunnett’s post hoc test. Asterisks denote significance: ** *p* < 0.01, *** *p* < 0.001, **** *p* < 0.0001 versus HCQ vehicle.

Increased reliance upon lysosomal processes such as autophagy for both energy production and evasion of chemotherapy-mediated cell death could provide an exploitable metabolic “weakness” when combined with cytotoxic agents. Thus, we next investigated whether triple therapy would increase cytotoxicity beyond that seen with dual treatment (i.e., with BMS-754807 + CP or HCQ + CP). TNBC cells were first treated with BMS-754807 for 24 h then with BMS-754807, CP, and HCQ (0–30 μM) for an additional 48 h. Concentrations of BMS-754807 and CP that produced high levels of synergy and cytotoxicity rates of ~50% were selected for each cell line (Table 1). In B6C3TAg 2.51, E0771, MDA-MB-231, and MDA-MB-468 cells, the triple-agent treatment significantly increased cytotoxicity 11–52% compared with BMS-754807 and CP combination treatment (Figure 5A–D).

To delineate the contribution of autophagy inhibition to the cytotoxic effects of HCQ treatment, we established TNBC cell lines expressing shRNA targeting the expression of ATG5 (essential for LC3 lipidation) or a non-targeting control. Altered sensitivity to BMS-754807 or CP was not evident upon suppression of ATG5 expression (Figure S4), indicating that the cytotoxic effects of HCQ are driven by disruption of lysosomal function, rather than strictly inhibition of autophagy.

### 3.5. BMS-754807 and HCQ Sensitize metM-Wnt^lung^ Tumors to CP

**Figure 6:**
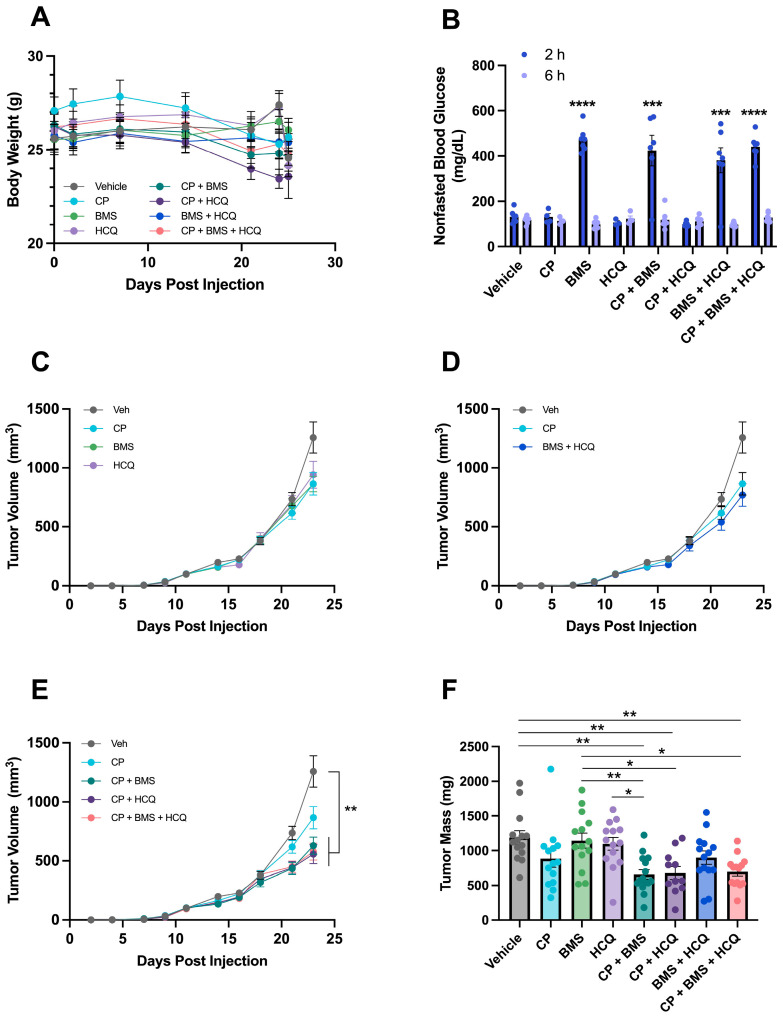
Separate and combined effects of BMS-754807, HCQ, and CP on tumor mass in a metM-Wnt^lung^ model of TNBC. (**A**) Body weight of mice treated with BMS-754807, CP, HCQ, or combinations; (**B**) blood glucose was assessed in all groups 2 h and 6 h after dosing with drug combinations; (**C**–**E**) tumor volume; and (**F**) tumor mass at endpoint in mice injected with metM-Wnt^lung^ cells. Data presented as mean ± SEM. (**B**) Effect of drug interventions on blood glucose was analyzed by unpaired *t*-test with * *p* < 0.05, ** *p* < 0.01, *** *p* < 0.001 and **** *p* < 0.0001. (**F**) Differences in final tumor mass across all groups were analyzed using three-way ANOVA and Tukey post hoc test. Different letters indicate significant differences between groups.

Previous findings suggested Wnt-driven mammary tumors in vivo are more sensitive to chemotherapy than Wnt-driven cell lines in vitro [38]. C57BL/6 mice bearing metM-Wnt^lung^ mammary tumors were treated with either vehicle, BMS-754807, HCQ, CP, or combinations. Body weights were generally stable over the duration of the study for each treatment group, suggesting treatment was not toxic (Figure 6A). After 23 d of drug treatment, mice dosed with BMS-754807 either alone or in combination with HCQ or CP showed increased blood glucose concentrations 2 h after dosing, indicating inhibition of IR, which was restored to the same levels as vehicle-treated mice 6 h after dosing (Figure 6B). Compared with vehicle, single agents (e.g., CP, BMS-754807, and HCQ) did not significantly alter tumor volume (Figure 6C) and, similarly, the combination of BMS-754807 and HCQ did not reduce tumor growth (Figure 6D). However, the combination of CP with either BMS-754807 or HCQ significantly decreased final tumor mass by 45% and 43%, respectively, compared with vehicle. Treatment with all three compounds reduced tumor mass by 41% compared with vehicle treatment, which was not different from dual treatment with CP plus either BMS-754807 or HCQ (Figure 6E). At study termination, tumor mass was significantly reduced, relative to vehicle, by all carboplatin-containing combination therapies but by no monotherapy (Figure 6F). 

## 4. Discussion

While targeted therapies exist for hormone receptor-positive BCs, a lack of targetable receptors leaves cytotoxic chemotherapy or pembrolizumab as the standard-of-care treatment for patients with TNBC [5,7]. A paradoxical response to chemotherapy exists in TNBC characterized by high initial chemosensitivity but higher relapse rates in those with residual disease [11,39]. Additionally, platinum-based chemotherapies are associated with serious and dose-limiting side effects [40,41,42]. Investigation into novel combinatorial strategies to minimize chemotherapy resistance is necessary to improve treatment outcomes, reduce the cumulative dose of platinum-based drugs, and prevent deaths from TNBC. Building upon our in vitro data, our in vivo findings showed that combining CP with either BMS-754807 or HCQ significantly reduced tumor burden in mice compared with vehicle-treated mice. Thus, our findings demonstrate that tandem IR/IGF-1R inhibition and disruption of lysosomal dynamics enhance the effects of carboplatin both in vitro and in vivo. Though we did not formally pursue quantification of toxicity following therapy, no differences were observed in bodyweight or mortality of study animals across treatment groups. Future studies should address the therapeutic index of these drugs when used in combination.

Our results further showed that, in a panel of murine and human TNBC cells [28,43,44,45], treatment with the IR/IGF-1R inhibitor BMS-754807 caused cytotoxicity across all cell lines, though some cell lines were more sensitive to the treatment than others (Figure 1). These results agree with other literature showing cytotoxic effects of BMS-754807 in other cancer types, including pancreatic, colon, leukemia, and non-small cell lung cancer [46,47]. Previous testing of BMS-754807 in a panel of 30 BC cell lines revealed that basal-like/TNBC cell lines were more sensitive to BMS-754807, while luminal/HER2 overexpressing cell lines showed greater resistance [48]. A preclinical study using a model of colon cancer showed that BMS-754807 treatment alone decreases tumor volume compared with vehicle treatment [49]. However, our results and others showed that BMS-754807 treatment alone does not affect mammary tumor size relative to vehicle treatment (Figure 6C,D) [31]. Despite encouraging in vitro and mixed animal model findings, clinical trials assessing the use of IR/IGF-1R inhibitors as monotherapy have proved ineffective at improving progression-free survival compared with chemotherapy. Thus, while promising effects of IR/IGF-1R inhibition are reported in preclinical studies, combining IR/IGF-1R inhibitors with additional therapeutics in the clinical setting may be necessary to achieve reductions in tumor size [25,26].

IR/IGF-1R signaling can mediate chemoresistance to platinum-based alkylating agents by supporting hyperactivation of proliferative pathways [24,50]. We found that inhibition of IR/IGF-1R signaling with BMS-754807 sensitized TNBC cells to CP, with increased cytotoxicity compared with CP treatment alone (Figure 3). A phase I/II clinical trial investigating linsitinib, another dual IR/IGF-1R inhibitor, in combination with the chemotherapy drug paclitaxel found that, compared with paclitaxel alone, adding intermittent or continuous linsitinib treatment did not improve overall survival, response rate, or disease control rate in platinum-resistant ovarian cancer [51]. Further translational efforts are required to determine if the benefits of dual treatment with an IR/IGF-1R inhibitor and chemotherapy observed in vitro can be achieved in patients with cancer.

To further define the relationship between BMS-754807 and CP, we evaluated synergy, which calculates the excess of observed-over-expected effect. Models for evaluating synergy for drug combination studies include the Loewe model, which is used when two drugs target the same pathway, and the Bliss model, which is used when two drugs target different signaling pathways [52]. Thus, for analysis of BMS-754807 and CP, we used the Bliss independence model, which assumes that the fractional response of two drugs in combination equals the sum of the two fractional responses of each drug minus their product [37]. We detected a synergistic relationship between BMS-754807 and CP in all tested TNBC cell lines (Table 1). In previous reports, BMS-754807 sensitized human breast cancer cells to cisplatin treatment through modulation of the DNA damage response pathway [53]. Furthermore, given that dietary modifications including calorie restriction and intermittent fasting decrease signaling through IR/IGF-1R, our data with BMS-754807 complement the previous literature, showing that fasting at the time of chemotherapy treatment not only reduces tumor size in mouse models but also mitigates treatment-induced side effects [54,55,56,57,58]. Concerns about the feasibility of implementing fasting-based diets are nontrivial and, thus, utilization of IR/IGF-1R inhibitors such as BMS-754807 may be a valuable alternative approach to block insulin signaling.

We next investigated the contribution of lysosomal processes to chemotherapy resistance in TNBC cells. We utilized HCQ, which has lower gastrointestinal and ocular toxicity than its parent compound chloroquine [59]. Sensitization of TNBC cells with HCQ followed by CP treatment resulted in increased cancer cell cytotoxicity compared with CP treatment alone (Figure 4). We found that combination treatment of HCQ and CP consistently produced either synergistic or additive effects across multiple TNBC cell lines (Table 2). These data agree with the previous literature in esophageal cancer cells in which combination treatment of cisplatin and chloroquine significantly reduced cell viability compared with cisplatin treatment alone [60]. Similarly, in a model of TNBC, CP and chloroquine combination treatment significantly reduced cell viability compared with CP treatment alone [61]. As such, the collective data suggest that disruption of lysosomal function may improve response to CP in cancer cells.

In addition to chemotherapy, inhibition of IR/IGF-1R regulates both autophagy and lysosomal function by limiting activation of the PI3K/AKT/mTOR cascade [62,63,64]. As such, we sought to determine if the cytotoxic effects of HCQ would be more pronounced in the presence of both BMS-754807 and CP in vitro. Our findings revealed that HCQ treatment in the presence of BMS-754807 and CP resulted in a modest increase in cytotoxicity relative to treatment with BMS-754807 and CP (Figure 5). Further, while BMS-754807 treatment promoted autophagy (Figure S3), suppressing ATG5 expression to inhibit autophagy without disrupting lysosomal function did not alter sensitivity to BMS-754807 or carboplatin, indicating that inhibiting autophagy alone was insufficient to explain the cytotoxic effects of HCQ (Figure S4). 

To further expand upon these findings, we investigated the effects of all three drugs in a murine model of TNBC utilizing the metM-Wnt^lung^ cell line. At the study endpoint, CP treatment alone had no significant effect on tumor mass (Figure 6). However, in agreement with our in vitro findings, the addition of either BMS-754807 or HCQ to CP therapy decreased tumor mass compared with vehicle treatment. These data indicate that the use of either inhibitor enhances response to chemotherapy and, thus, may have important implications for improving treatment response in patients with cancer. Similar results were previously reported in mice with xenograft injections of SUM159 breast cancer cells in which CP treatment alone did not affect tumor growth rate, but treatment with both CP and chloroquine significantly reduced tumor growth rates [61].

The previous literature shows benefits of combining inhibition of IR/IGF-1R and autophagy. For example, BMS-754807 increased autophagic flux in pancreatic cancer cells and dual treatment with BMS-754807 and chloroquine decreased cancer cell viability and tumor growth [31]. Similarly, a PI3K inhibitor increased autophagy in head and neck squamous cell carcinoma and combining the PI3K inhibitor with chloroquine decreased cell viability in a synergistic fashion [65]. However, results from our tumor study and in vitro work do not show a benefit of targeting these two pathways simultaneously in a model of TNBC (Figure 5 and Figure 6). Future research is needed to determine the effects of BMS-754807 on autophagic flux in breast cancer cells to determine if the BMS-754807-mediated increase in autophagy reported in pancreatic cancer [31] and head and neck cancers [65] is conserved across cancer types.

## 5. Conclusions

Given the growing prevalence of BC and current lack of targeted therapies against TNBC, new strategies to minimize chemotherapy resistance are urgently needed. Here, we have shown that metabolic modulation targeting IR/IGF-1R signaling and lysosomal function enhances the effects of CP both in vitro and in vivo. Our in vitro data suggest that the use of metabolic modulators co-operate in a synergistic or additive fashion with CP in reducing TNBC cell viability. In a mouse model of TNBC, combining CP with either BMS-754807 or HCQ significantly reduced tumor size. Thus, we conclude that CP sensitivity can be enhanced by metabolically targeted therapies. Further work is required to gain deeper understanding of the mechanisms underlying the cytotoxic effects of exogenous metabolic modulators in combination with chemotherapy to inhibit TNBC progression.

## Figures and Tables

**Table 1:** Bliss independence synergy scores following treatment of TNBC cells with BMS-754807 and CP from *n* = 3 (E0771, MDA-MB-231, MDA-MB-468) and *n* = 4 (B6C3TAg 2.51, metM-Wnt^lung^) experiments.

Cell Line	BMS-754807 (µM)	CP (µg/mL)	Synergy Score	Cytotoxicity (%)	Highest Synergy Score
E0771	1.25	25	12.74	47.9	15.77
B6C3TAg 2.51	1.25	12.5	34.71	56.0	34.71
metM-Wnt^lung^	1.25	25	14.19	58.8	14.50
MDA-MB-231	1.25	200	13.98	56.8	13.98
MDA-MB-468	0.625	12.5	14.87	53.5	14.87

**Table 2:** Bliss independence synergy scores following treatment of TNBC cells with HCQ and CP for *n* = 3 (B6C3TAg 2.51, E0771, MDA-MB-231, MDA-MB-468) and *n* = 4 (metM-Wnt^lung^) experiments.

Cell Line	HCQ (µM)	CP (µg/mL)	Highest Synergy Score	Cytotoxicity (%)
E0771	15	50	2.67	91.3
B6C3TAg 2.51	2.5	25	0.67	85.0
metM-Wnt^lung^	5	25	8.71	69.7
MDA-MB-231	5	200	12.42	41.1
MDA-MB-468	3.75	12.5	15.07	60.0

## Data Availability

All data are available upon reasonable request to the corresponding author.

## Supplementary Materials

The following supporting information can be downloaded at: https://www.mdpi.com/article/10.3390/cells13100806/s1, Figure S1: Effect of BMS-754807 on proteins downstream of IR/IGF-1R; Figure S2: Effect of HCQ treatment on cytotoxicity in TNBC cells; Figure S3: LC3 lipidation following BMS-754807 treatment; Figure S4: Effect of ATG5 depletion on BMS-754807 and carboplatin sensitivity.
