# Supplementary material for: Exogenous Metabolic Modulators Improve Response to Carboplatin in Triple-Negative Breast Cancer
Source: Cells. 2024 May 9;13(10):806. doi: 10.3390/cells13100806 (PMC11119195; doi:10.3390/cells13100806)
Supplement: Supplementary file 1 [file cells-13-00806-s001.zip › cells-2953817-supplementary.pdf]

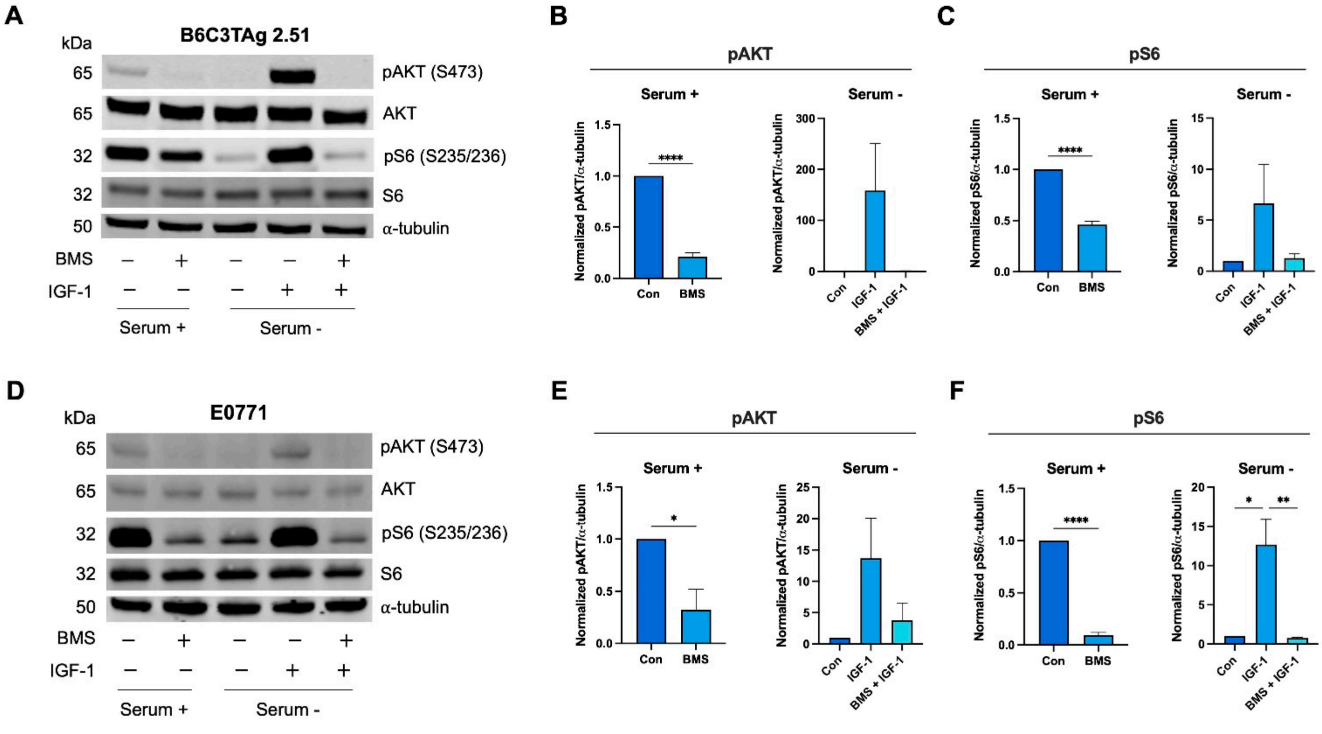

Supplemental Figure S1. Effect of BMS-754807 on proteins downstream of IR/IGF-1R. Representative western blots from **(A)** B6C3TAg 2.51 and **(D)** E0771 cells and quantification of **(B, E)** pAKT and **(C, F)** pS6 relative to AKT and a-tubulin or S6 and a-tubulin, respectively. Data are presented as mean ± SEM from n=3 experimental replicates. Differences between two groups were analyzed using unpaired t-test; differences across groups were analyzed using one-way ANOVA, followed by Tukey's post hoc test. Asterisks denote significance: *P<0.05, **P<0.01, ****P<0.0001 versus vehicle.

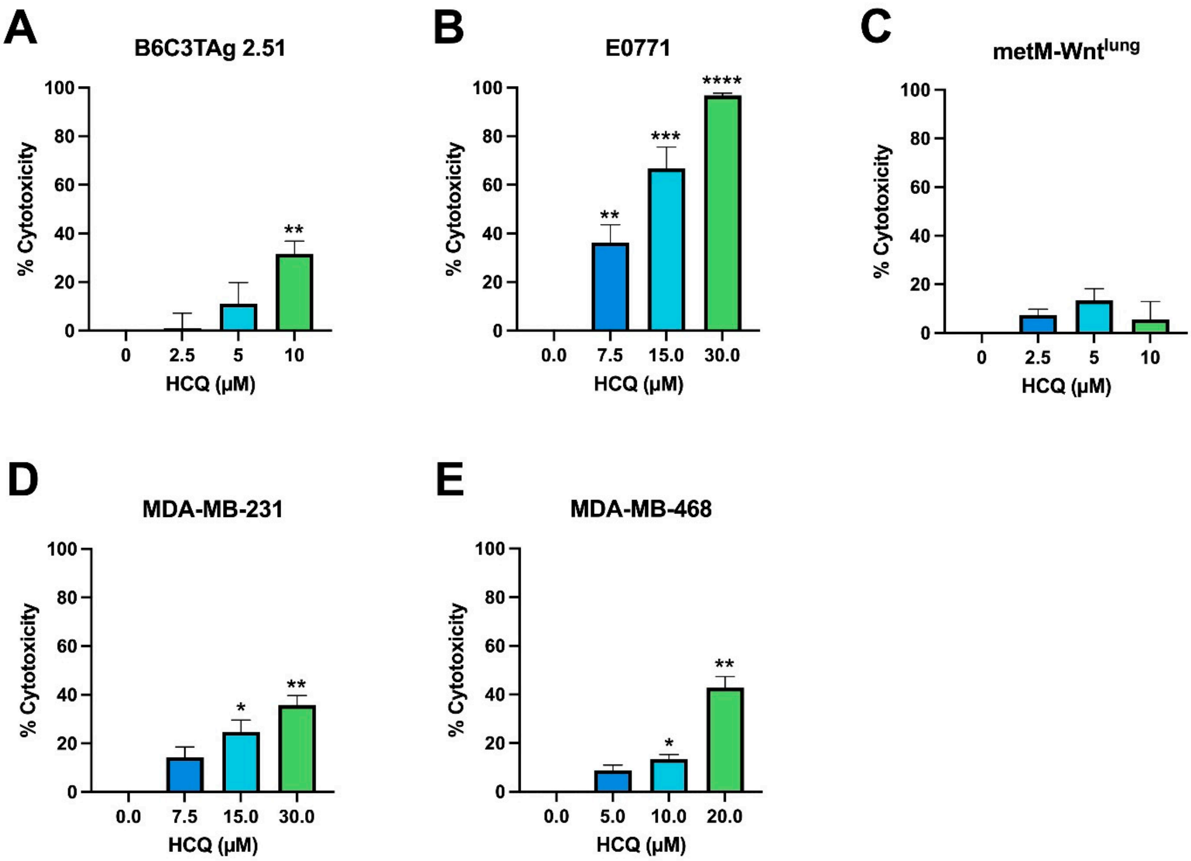

Supplemental Figure S2. Effect of HCQ treatment on cytotoxicity in TNBC cells. Cytotoxicity following 48h treatment with HCQ was assessed in **(A)** B6C3TAg 2.51; **(B)** E0771; **(C)** metM-Wnt^{lung}; **(D)** MDA-MB-231; **(E)** MDA-MB-468 TNBC cell lines by MTT assay. Data presented as mean ± SEM for n=3 (metM-Wnt^{lung}), n=4 (MDA-MB-231, MDA-MB-468), n=5 (B6C3TAg 2.51), n=9 (E0771) experiments. Differences between cells exposed to more than two experimental conditions were analyzed using one-way ANOVA, followed by Dunnett's post hoc test. Asterisks denote significance: *P<0.05, **P<0.01, ***P<0.001, ****P<0.0001 versus vehicle.

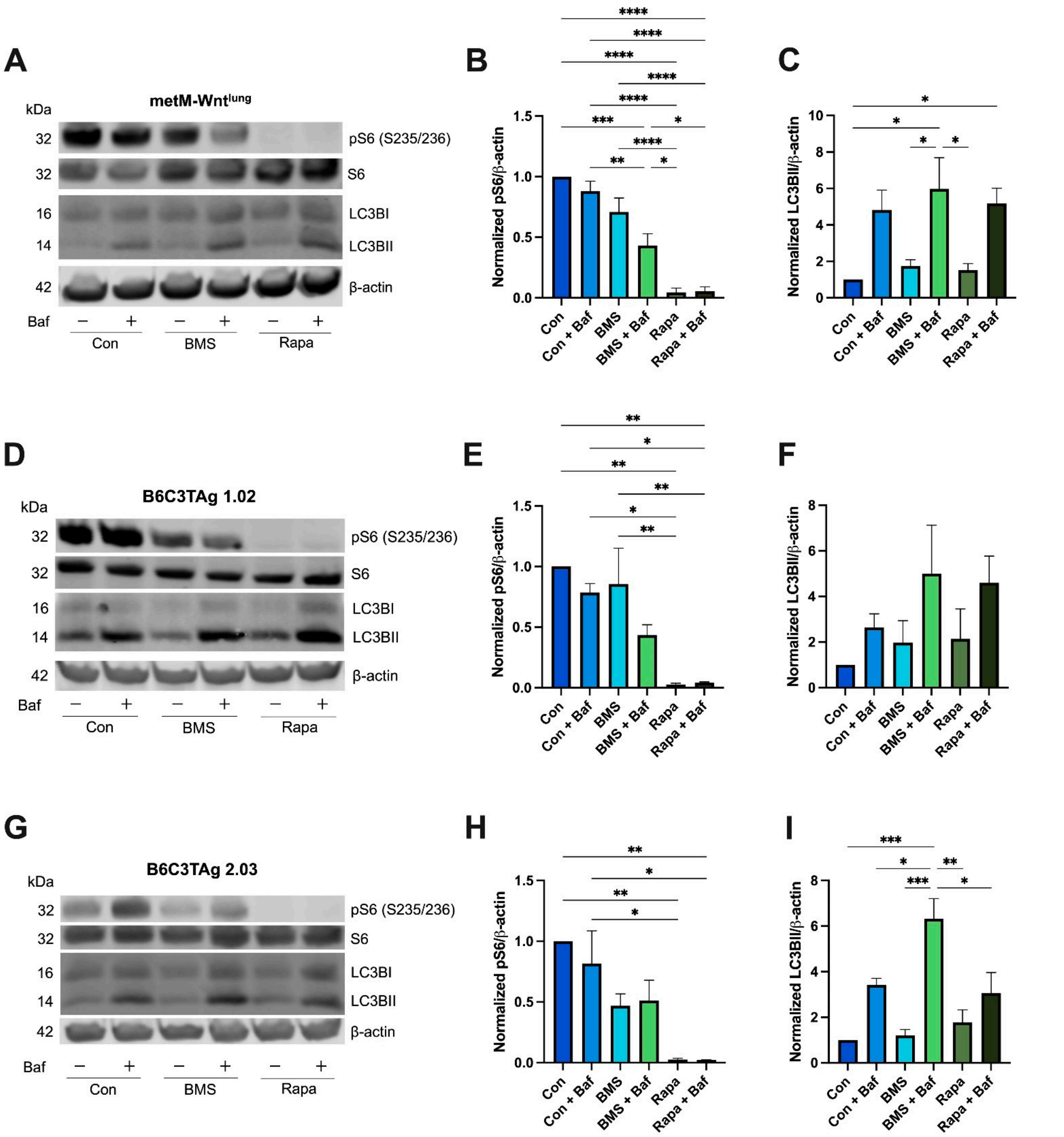

Supplemental Figure S3. LC3 lipidation following BMS-754807 treatment. Lipidation of LC3 (LC3-II) following 4h pretreatment with 1μM BMS-754807 or rapamycin followed by treatment with or without 200nM bafilomycin A1 (Baf) for 4h in **(A-C)** metM-Wnt^{lung}; **(D-F)** B6C3TAg 1.02; **(G-I)** and 2.03. Data presented as mean ± SEM for n=4 (A-C) and n=3 (D-I) experiments. Differences between cells exposed to more than two experimental conditions were analyzed using one-way ANOVA, followed by Tukey's post hoc test. Asterisks denote significance: *P<0.05, **P<0.01, ***P<0.001, ****P<0.0001

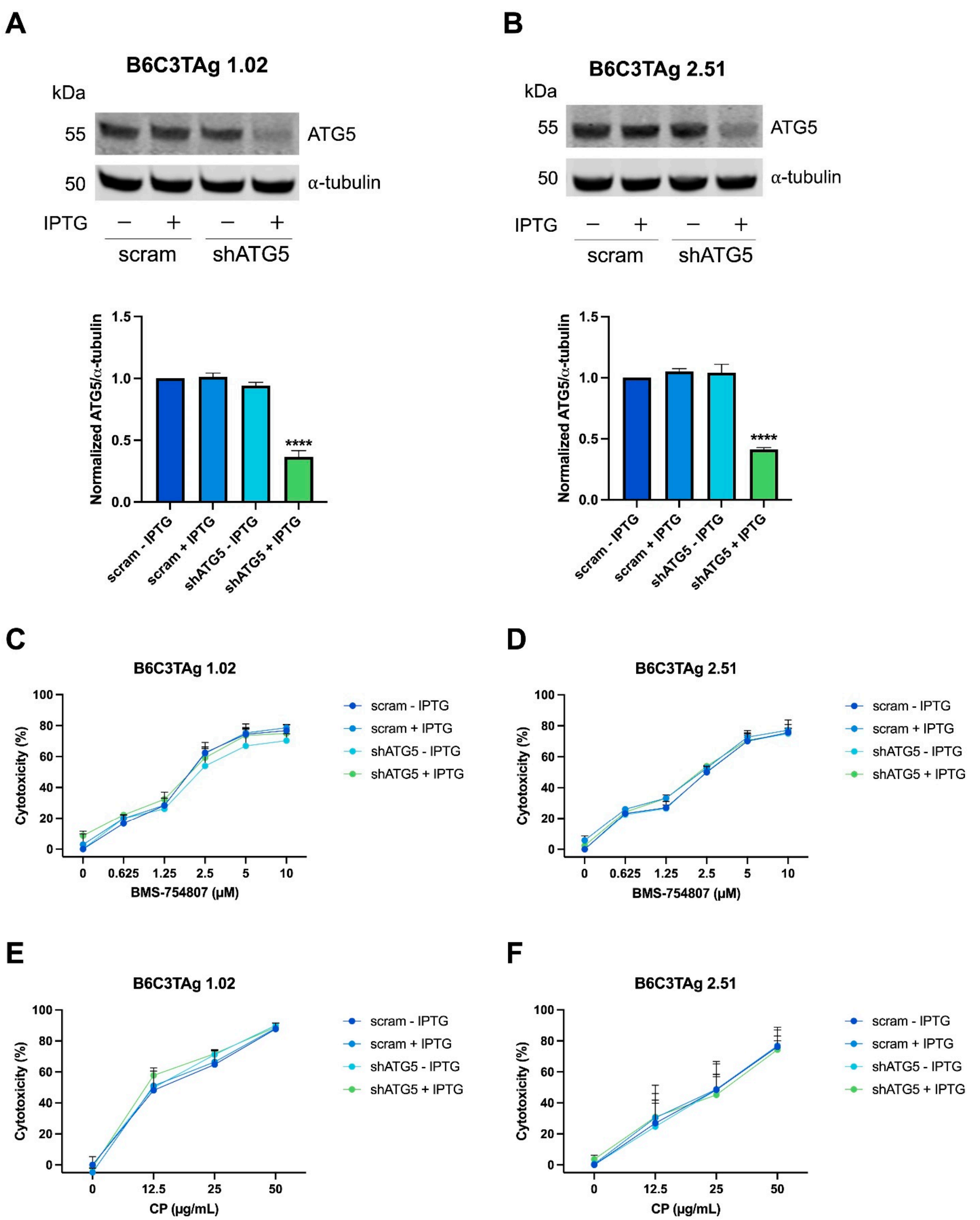

Supplemental Figure S4. Effect of ATG5 depletion on BMS-754807 and carboplatin sensitivity. B6C3TAg 1.02 and 2.51 cells were transduced with IPTG-inducible shRNA targeting ATG5 or a scramble control. **(A-B)** Suppression of ATG5 with 48h 1mM IPTG treatment. **(C-D)** Cytotoxicity following 48h treatment with 1mM IPTG and indicated concentrations of BMS-754807 by MTT assay. **(E-F)** Cytotoxicity following 48h treatment with 1mM IPTG and indicated concentrations of carboplatin by MTT assay. Data presented as mean ± SEM for n=4 (A-B) and n=2 (C-F) experiments. Differences between cells exposed to more than two experimental conditions were analyzed using one-way ANOVA (A-B), followed by Dunnett's post hoc test. Asterisks denote significance: ****P<0.0001 versus scram - IPTG.
